# Opportunities for Promoting Physical Activity in Rural Communities by Understanding the Interests and Values of Community Members

**DOI:** 10.1155/2017/8608432

**Published:** 2017-08-08

**Authors:** Thomas Park, Amy A. Eyler, Rachel G. Tabak, Cheryl Valko, Ross C. Brownson

**Affiliations:** ^1^Barnes-Jewish Hospital, Washington University School of Medicine, St. Louis, MO 63108, USA; ^2^Prevention Research Center in St. Louis, Brown School, Washington University in St. Louis, St. Louis, MO 63130, USA; ^3^Division of Public Health Sciences and Alvin J. Siteman Cancer Center, Washington University School of Medicine, Washington University in St. Louis, St. Louis, MO, USA

## Abstract

**Purpose:**

Physical activity (PA) has well-established health benefits, but most Americans do not meet national guidelines. In southeastern Missouri, trails have been developed to increase rates of PA. Although this has had success, broad-scale interventions will be needed to improve rates further. In this study, we surveyed residents of southeastern Missouri to identify ways to improve rates of PA.

**Methods:**

We conducted a telephone survey in 2015 of adults (*n* = 524) from eight rural Missouri towns that had walking trails, regarding their activities and interests.

**Findings:**

Forty percent of respondents reported both walking and meeting PA recommendations, 29% reported walking but not meeting PA recommendations, and the remainder did not walk or did not answer. Respondents who used the trails were significantly more likely to meet PA recommendations (odds ratio = 2.7; 95% confidence interval = 1.7, 4.5). Certain values and interests that may encourage PA or draw people to trails were common.

**Conclusions:**

The group that walked but did not meet PA recommendations would be the ideal group to target for intervention, which could focus on their reported values and interests (e.g., personal relationships, being outdoors). Use of walking trails was associated with meeting PA recommendations.

## 1. Introduction

It is well established that people who are more physically active have a lower risk of chronic diseases than those who are less active [1]. The Physical Activity Guidelines Advisory Committee currently recommends at least 150 minutes of moderate-intensity aerobic physical activity per week for adults to obtain substantial health benefits [2]. However, a significant proportion of adults in the United States do not adhere to these recommendations [3]. Despite these well-established health benefits of regular physical activity (PA), only 51.7% of adults met the national Physical Activity Guidelines for Americans in 2016 [4].

Rural residents are disproportionally affected by rates of physical inactivity, including lower levels of PA in leisure time [5, 6]. This may be due in part to limited walkability, as rural communities often lack built environment features such as sidewalks and parks [7, 8]. Neighborhoods with higher walkability scores are associated with more walking, less obesity, and lower coronary heart disease risk [9]. Over the past two decades, our team has been involved with constructing 40 walking trails in rural southeast Missouri to increase access to and promote PA among residents [7, 10]. After the development of the trails, PA and cholesterol screenings increased in the region, suggesting possible reduction in cardiovascular disease risk [10].

These health benefits may incentivize a person's decision to increase PA, but life priorities and values other than health can also influence behavioral change. For example, as described in the Self-Determination Theory, when the social environment is optimal, individuals become more intrinsically motivated [11]. Social values can influence behavior. When applied to PA, this theory postulates that health behavior interventions can target individuals' interests to help adopt and maintain an active lifestyle [11]. For example, if people find being out in nature fulfilling or like exercising with friends, they may be more likely to engage in PA with others or outdoors [12].

Although the trails themselves appear to increase PA in the community, an opportunity exists to implement broad-scale interventions with the aim of increasing trail use among residents [13]. The purpose of this study was to survey adults in the Bootheel and Ozark regions of southeastern Missouri to help identify novel approaches for increasing the rate of PA in rural communities.

## 2. Methods

The 2013 Rural-Urban Continuum Codes (RUCC) form a classification scheme that distinguishes metropolitan counties by the population size of their metropolitan area and nonmetropolitan counties by the degree of urbanization and adjacency to a metropolitan area using a scale from 1 (metropolitan) to 9 (completely rural) [14]. Much of southeastern Missouri is considered rural, with counties in the Ozark and Bootheel regions being rated as between 7 and 9 RUCC [14]. These regions, in particular, have limited opportunities for PA and received new walking trails as part of the Bootheel Heart Health Project [10]. This study takes place in eight towns where trails were developed from this project [10]. Details regarding the development of the trails and the trail characteristics have been discussed previously by Wiggs et al. [13]. As noted previously [13], the majority of trails were located in residential park areas within city limits. They are generally asphalt (65%), gravel (24%), or wood chip (11%) covered. The eight communities were chosen based on population, race, and prevalence of obesity. We also consulted with a local expert in southeast Missouri who participated in the development of the trails to confirm the most appropriate areas to assess to cover a range of settings and conditions. This study was given exempt status by the institutional review board (IRB) at Washington University in St. Louis and subjects were provided an exempt information sheet prior to participation.

An exploratory study was conducted in May through June 2015, where residents from eight rural Missouri towns were interviewed by telephone. A survey research firm (Survey Research Laboratory, Mississippi State University) was contracted to conduct the telephone survey. A list of random phone numbers was matched to target the specific geographic areas of interest. A dual-frame (cellphone and landline) sampling design was used to maximize coverage of eligible respondents. To ensure a reproducible and representative sample, probability-based sampling via random digit dial (RDD) was utilized within each of the two frames.

The survey consisted of several sections. In section A, respondents were asked about their age and location. In section B, respondents rated interests (e.g., volunteering) and values (e.g., personal health) on a scale from 1 (not at all enjoyable) to 5 (very enjoyable) for interests and from 1 (not very important) to 4 (very important) or 1 (strongly disagree) to 5 (strongly agree) for values. In section C, respondents were asked about their local walking trail awareness, access, and use. This section also included a qualitative portion where respondents could describe what activity or events would encourage visiting the trails. In section D, respondents were asked about their activity level. This section was used to determine if participants walked and met PA recommendations for aerobic activity (walks and does 150 minutes of PA per week), walked but did not meet PA recommendations for aerobic activity (walks but does not meet 150 minutes of PA per week), or did not walk. In section E, respondents were asked about cellphone use. In section F, respondents were asked about their demographics. The data were analyzed with SPSS, first with descriptive statistics and next with odds ratios using multinomial logistic regressions and 95% confidence intervals (unadjusted).

## 3. Results

Among eligible respondents, the response rate was 35%. Of the total completed surveys (524 out of 571, cooperation rate of 91.8%), 196 (37%) respondents were surveyed over cellphone and 328 (63%) were surveyed over landline. The majority of respondents were above 50, female, high school educated, and Caucasian. Most of the participants (63%) were either overweight or obese. Of the participants, 40% reported walking and also meeting PA recommendations, 29% reported walking and not meeting PA recommendations, and the remainder either did not walk (23%) or did not respond to the question (9%). Of those surveyed, 65% were aware of the trail, and 34% have used the trails at some point, and 14% have used the trail often/very often.

The participants who often/very often used the trails were above 55, female, and Caucasian, similar to the baseline characteristics of those surveyed (Table 1). However, there were a disproportionate number of people who had normal BMI and used the trails often/very often. Of those who were aware of the trails, 42% used them very often/often. Individuals who used the trails were significantly more likely to meet PA recommendations (odds ratio = 2.7; 95% confidence interval = 1.7, 4.5).

Those respondents who walked but did not meet PA recommendations were a particular focus for the study. Participants who walk but not enough to meet PA recommendations were also similar to the overall population that was surveyed: above 55, female, Caucasian, and overweight. Those that walked but did not meet PA recommendations reported that they place high value on relationships with family and friends, their own health, PA, and being outdoors/in nature (Table 2). They also reported enjoying listening to music, reading, watching TV/movies, cooking, and yardwork.

We also collected qualitative responses about events or activities that would encourage trail use. Community events (e.g., picnics, races or walking events, festivals, and kid-friendly activities; 17%), sporting events (5.5%), and social organizations (e.g., group walks or walking partners; 3.2%) were among suggestions. Also, 38% of respondents aware of nearby trails found out about the trail because they “happened to see it one day,” while 20% heard about it from a friend. When asked about barriers, 13% of respondents said the trail was too far away from home, while 12% said lack of interest kept them from using the trail.

## 4. Discussion

Community trails are a way to provide equitable and accessible opportunities for PA in rural areas where few other opportunities exist. In our study, a significant percentage of respondents in communities with trails have never used the trails (32%). However, our results also indicate that individuals who used the trails had significantly greater odds of meeting PA recommendations (OR = 2.7). This finding is consistent with other studies [12, 15–17]. Interventions to increase the utilization of existing trails in rural communities have the potential to increase PA and positively impact population cardiovascular disease risk.

Almost one-third of survey respondents reported doing some walking, but not meeting the PA recommendation. Based on the transtheoretical model, which uses stages of change to predict behavior, this group may be the most amenable to interventions to increase trail utilization [18]. These respondents likely fall within the contemplation or the preparation stage, meaning they are more aware of personal consequences and potential benefits and are preparing to make a behavior change in the foreseeable future. In contrast, those who did not report walking likely fit into the precontemplation stage, in which people are not planning to take action in the near future and are therefore less likely to respond to intervention.

Developing effective strategies to encourage this group to walk more than they currently do may involve identifying key values and priorities and integrating them into a multilevel intervention approach as described by the social-ecological model [19]. This group reported to value relationships with family and friends, their own health, PA, and being outdoors/in nature. These values and activities could be utilized as intervention leverage points and build on multilevel strategies, which have had some evidence of success in the literature [19]. For example, individual level intervention components could highlight reinforcing or increasing knowledge about the health benefits of PA, including walking or goal setting. Interpersonal-level components could build on the value of family and friend support by facilitating walking groups [20]. Community-level strategies may include promotion and implementation of different types of community events at the trails (e.g., fishing). Since nearly 40% of respondents just “happened to see the trail one day,” it is likely that more local, multilevel promotion efforts are needed to publicize the trails. This publicity would be consistent with the* Community Guide* recommendation to enhance or create opportunities for PA with outreach [21].

Furthermore, promotion of PA by using the trails can target multiple interests and be combined with other land uses. The majority of these trails are in residential park areas [13], which adheres to guidelines to combine pedestrian infrastructure with parks and recreational facility access [22]. If the location of the trails permits, they can be promoted as a way to connect homes, neighborhoods, stores, and facilities. Additionally, the trails can be promoted as a social gathering place (e.g., for seniors, families, and children and religious groups), a place to improve health and engage in physical activity (e.g., for fitness groups), and a place to enjoy nature (e.g., for schools, youth groups). Promotional events that target these interests may encourage the target group to walk more (e.g., family day, charity walks and races, competitive races or events, health/physical activity walks, and nature walks). Those who valued community engagement and volunteering could respond to promotion that focuses on improving the trails (e.g., beautification, maintenance) to further benefit the community by improving aesthetics, public perception of the space, and increasing home values [22].

Open-ended questions suggested that the population would be amenable to community events, sporting events, and social organizations. Our data also showed that, of those who were aware of the trails in the area, 42% used the trails very often/often. Consistent with previous publications [23], this suggests that outreach and increasing awareness of the trails' existence would also be a valid intervention. Awareness could be enhanced through various channels such as social media, print, and other strategies identified by community stakeholders as applicable.

The limitations of this study include the cross-sectional design using unadjusted models and limited generalizability of the data obtained from a region in a specific state. Also, the study relies on self-report by the participants (e.g., height, weight, amount of PA, and trail use) and does not explore what other PA are done other than walking, which types of trails they prefer, or what activities they do when they visit the trails. In spite of these limitations, this study helps to identify values and priorities of a rural population to inform a physical activity intervention. Future studies could use more reliable data collection methods (e.g., accelerometers, GPS tracking) longitudinally to improve the quality of the data.

In conclusion, this survey of residents of eight towns in rural southeast Missouri highlighted a nearly threefold increase in PA for residents who use trails. This study also evaluated participants' values and interests that could be targeted for a multilevel intervention designed to encourage the use of walking trails, particularly among residents who reported walking but did not meet PA recommendations. A rigorous evaluation of multilevel intervention strategies will also add to the evidence of best practices to increase PA in rural communities.

## Figures and Tables

**(a):** 

	Trail use		
	Very often/often (%)	Sometimes (%)	Rarely (%)
Age			
18 to 34	15 (50)	13 (43)	2 (7)
35 to 54	16 (32)	21 (42)	13 (26)
>55	37 (43)	34 (40)	15 (17)

Age total	68 (41)	68 (41)	30 (18)

Sex			
Male	26 (42)	25 (40)	11 (18)
Female	48 (42)	44 (39)	21 (19)

Sex total	74 (42)	69 (39)	32 (18)

Race			
Caucasian	58 (41)	57 (40)	26 (18)
African-American	10 (50)	9 (40)	2 (10)
Other	4 (80)	1 (20)	0

Race total	72 (43)	67 (40)	28 (17)

BMI			
Underweight	3 (75)	1 (25)	0
Normal	33 (63)	12 (23)	7 (13)
Overweight	17 (28)	29 (48)	14 (23)
Obese	11 (28)	19 (48)	10 (25)

BMI total	64 (41)	61 (39)	31 (20)

Aware of trails			
Yes	74 (42)	70 (40)	32 (18)
No	0	0	0

Trail awareness total	74 (42)	70 (40)	32 (18)

**(b):** 

	Physical activity		
	Does not walk (%)	Walks/fails PA recs. (%)	Walks/meets PA recs. (%)
Age			
18 to 34	7 (11)	17 (26)	41 (63)
35 to 54	20 (19)	32 (30)	54 (51)
>55	83 (30)	95 (34)	103 (37)

Age total	110 (24)	144 (32)	198 (44)

Sex			
Male	41 (25)	42 (26)	80 (49)
Female	78 (25)	110 (35)	125 (40)

Sex total	119 (25)	152 (32)	205 (43)

Race			
Caucasian	96 (25)	126 (32)	168 (43)
African-American	13 (25)	12 (23)	27 (52)
Other	4 (22)	8 (44)	6 (33)

Race total	113 (25)	146 (32)	201 (44)

BMI			
Underweight	3 (33)	1 (11)	5 (56)
Normal	25 (21)	41 (34)	54 (45)
Overweight	37 (22)	56 (33)	77 (45)
Obese	39 (30)	39 (30)	51 (40)

BMI total	104 (24)	137 (32)	187 (44)

Aware of trails			
Yes	73 (23)	106 (34)	133 (42)
No	46 (28)	47 (28)	72 (44)

Trail awareness total	119 (25)	153 (32)	205 (43)

**Table 2:** Life pursuits/values enjoyed by those who walk but do not meet physical activity recommendations (*n* = 153).

	Level of enjoyment/importance			
	Enjoyable/important (%)	Neutral (%)	Unenjoyable/unimportant (%)	Unknown (%)
Relationships with family/relatives	146 (95)	3 (2)	4 (3)	0
Own health	144 (94)	7 (5)	2 (1)	0
Relationships with friends	139 (91)	10 (7)	3 (2)	1 (1)
Physical activity	120 (78)	22 (14)	11 (7)	0
Being outdoors and in nature	119 (78)	26 (17)	7 (5)	1 (1)
Living a long life	114 (75)	22 (14)	15 (10)	2 (1)
Social life/leisure activities	105 (69)	32 (21)	15 (10)	1 (1)
Being engaged in my community	104 (68)	28 (18)	17 (11)	4 (3)
Volunteering	95 (62)	29 (19)	26 (17)	3 (2)
Physical attractiveness	73 (48)	51 (33)	29 (19)	0
